# The Relationship Between Information Sources, Health Literacy, and COVID-19 Knowledge in the COVID-19 Infodemic: Cross-sectional Online Study in Japan

**DOI:** 10.2196/38332

**Published:** 2022-07-22

**Authors:** Mami Inoue, Kanako Shimoura, Momoko Nagai-Tanima, Tomoki Aoyama

**Affiliations:** 1 Department of Human Health Sciences Graduate School of Medicine Kyoto University Kyoto Japan

**Keywords:** COVID-19 infodemic, information source, health literacy, COVID-19 knowledge, social media, cross-sectional study, mass media, digital media

## Abstract

**Background:**

The COVID-19 pandemic has caused not only a disease epidemic but also an infodemic. Due to the increased use of the internet and social media, along with the development of communication technology, information has spread faster and farther during the COVID-19 infodemic. Moreover, the increased choice of information sources has made it more difficult to make sound decisions regarding information. Although social media is the most common source of misinformation, other forms of media can also spread misinformation. However, the media sources used by people with high health literacy and COVID-19 knowledge to obtain information are unclear. Furthermore, the association between the use of multiple information sources and health literacy or COVID-19 knowledge is ill-defined.

**Objective:**

This study aims to examine the following 3 aspects regarding the COVID-19 infodemic: (1) the relationship between health literacy, COVID-19 knowledge, and the number of information sources used; (2) the impact of media use on health literacy; and (3) the impact of media use on COVID-19 knowledge.

**Methods:**

An online cross-sectional study was conducted in November 2021. Participants were 477 individuals aged 20-69 years. After obtaining consent to participate in the study, participants were asked about sociodemographic indicators, sources of health-related information, health literacy, and COVID-19 knowledge. Sources of health-related information were categorized into 4 types: mass media, digital media, social media, and face-to-face communication. The Spearman rank correlation test was conducted to determine the relationship between health literacy, the number of correct answers to COVID-19 knowledge, and the number of information sources used. Multiple regression analysis was conducted with health literacy and the number of correct answers as dependent variables, the 4 media types as independent variables, and age and sex as adjustment variables.

**Results:**

Mass media was the most frequently used source of information, followed by digital media, face-to-face communication, and social media. Social media use was significantly higher among individuals aged 20-29 years than among other age groups. Significant positive correlations were found between health literacy, the number of positive responses to COVID-19 knowledge, and the number of information sources used. Multiple linear regression analysis showed that health literacy is associated with access to information from digital media and face-to-face communication. Additionally, COVID-19 knowledge was associated with access to information from mass media, digital media, and face-to-face communication.

**Conclusions:**

Health literacy and COVID-19 knowledge could be improved using diverse information sources, especially by providing opportunities to use digital media and face-to-face communication. Furthermore, it may be important to improve health literacy and provide accurate knowledge about COVID-19 to young adults.

## Introduction

The World Health Organization (WHO) declared COVID-19 as an infodemic at the Munich Security Conference in February 2020 [1]. An infodemic is a situation in which people are confused owing to a large amount of misinformation or false information during a disease outbreak [2]. The spread of misinformation during disease outbreaks occurred even during the Middle Ages [3]. However, this phenomenon is now amplified by the development of internet communication technology and the widespread use of social media. During the COVID-19 pandemic, TikTok reported a 38% increase in users, while Facebook and Twitter reported an almost 8% increase in users [4]. Consequently, the information spread much faster and farther away. Fake news and misinformation regarding COVID-19 have varied and have been confirmed multiple times [5]. For example, conspiracy theories state that COVID-19 is being spread by 5G communication technology and that the pandemic is an elaborate hoax spread mainly through Twitter [6,7]. Previous studies have reported that misinformation about COVID-19 spread rapidly on social media, including Facebook and YouTube [8]. Furthermore, a study examining the spread of 126,000 untruthful news stories over an 11-year period via Twitter found that misinformation was shared 70% more often than true information [9]. Social media is considered a prime source of misinformation because anyone, not just experts, can disseminate information.

However, examples of misinformation spread through sources other than social media have also been reported, such as the fake news about the shortage of toilet paper due to the COVID-19 pandemic since most toilet papers are made in China. This news spread in Japan in February and March 2020 [10]. When asked where they received this information, nearly 60% of the participants stated that television (TV) was the most common source [11]. In fact, the source of this fake news was social media, but it had not spread that far. However, it spread rapidly once it was picked up by TV programs and news sites. Therefore, any information source, not just social media, can be considered a potential source of misinformation.

Numerous sources of information are available. Information sources have been identified in previous studies and in the white paper on information and communications in Japan [11-13]. The major sources of information fall into 4 categories: face-to-face communication, such as conversations with family and friends; mass media, such as TV and newspapers; digital media, such as internet searches and news sites; and social media, such as Twitter, Facebook, and YouTube.

Face-to-face communication is a means of accessing information through conversation with others. Previous studies have reported that medical professionals, family members, and friends are sources of information [12-14]. Most people obtain health-related information from health care professionals [12-14]. Mass media is the traditional method of accessing information. Mass media refers to media that conveys information in a public, indirect, or 1-way manner, such as TV, radio, newspapers, magazines, and public relations materials. However, with the development of communication technology, the use of mass media has declined. In a 2014 survey, 70% of participants aged between 10 and 69 years obtained information through mass media [15]. However, in a 2021 survey, access to information through mass media decreased to less than 50% [11]. In the place of mass media, access to information through the internet is gaining ground. Digitized information obtained through the internet is referred to as digital media. Examples include the use of search engines, browsing webpages, and applications. Digital media also include social media. Social media is described as information that can be easily transmitted and exchanged or content that can be created and exchanged by anyone using the internet [16,17]. Social media is a digital medium that allows 2-way communication between individuals. Because people access information through a combination of face-to-face communication, mass media, digital media, and social media, determining the true information is considered more complex.

Health literacy is important in determining true information, especially in the context of infodemics [18,19]. Health literacy is the ability to access, understand, evaluate, and use information and services to promote and maintain health and well-being [20].

A previous study conducted in Australia reported that people with low health literacy have more difficulty in finding and understanding information about COVID-19 than those with high health literacy [21]. Health literacy has been reported to be positively correlated with the frequency of information-seeking behavior and the number of information sources used [22-24]. Thus, people with high health literacy are likely to have a higher frequency of information-seeking behavior, obtain information from multiple information media, and thus have higher disease knowledge. However, the relationship between health literacy, COVID-19 knowledge, and the number and types of information sources used has not been examined.

A previous study examined the use of 25 different information sources and found that highly health-literate people use medical websites and are less likely to use TV, social media, and blogs [12]. However, these 25 sources were too fragmented to examine the relationship between health literacy and multiple information sources. Another study of parents of children with asthma examined the relationship between 5 types of information sources: health professionals, family and friends, the internet, nonprint media, and print media. It was found that individuals with high health literacy obtain information from family, friends, and the internet [23]. However, the number of information sources was small and not exhaustive. Therefore, we thought it might be helpful to categorize information sources into 4 types in order to better capture the relationship and importance of multiple media to health literacy. The 4 types of media include face-to-face communication, after obtaining an exhaustive list of information sources. Furthermore, as mentioned earlier, misinformation about COVID-19 often originates from social media but may spread through other media. Therefore, it is essential to determine which media helps individuals to understand the information appropriately. Previous studies have examined COVID-19 knowledge and information sources (including 4 types of media) among university students in Jordan and Germany; however, knowledge and information sources were considered separately, and the relationship between them was not identified [22,25]. Several studies have examined the relationship between social media use and COVID-19 knowledge. A study conducted in China examined the relationship between social media use, eHealth literacy, and COVID-19 knowledge and found a positive correlation [26]. However, a study conducted in the United States reported that social media use is positively correlated with trust in misinformation about COVID-19 [27]. Furthermore, a study conducted in Canada reported that social media users misinterpret information about COVID-19 more frequently on social media and traditional news sites [28]; therefore, a unified view has not been reached. Moreover, these studies were limited to information obtained from social media and the internet and did not examine other information sources.

Therefore, this study aims to examine the following during the COVID-19 infodemic:

- The relationship between health literacy, COVID-19 knowledge, and the number of information sources used
- The influence of media use on health literacy
- The influence of media use on COVID-19 knowledge

The results may be used to indicate ways to spread true information to a larger population to manage the infodemic.

## Methods

### Study Design and Recruitment

A cross-sectional online survey was conducted from November 1 to 5, 2021, among individuals aged 20–69 years. Participants were recruited online by Surveroid (Marketing Applications Inc.). The number of participants by sex and age (20-29, 30-39, 40-49, 50-59, and 60-69 years) was set to be the same, and responses were accepted in the order of receipt. The purpose of the study was explained at the beginning of the online questionnaire survey. The submission of the online questionnaire implied consent to participate in the study. The online questionnaires were collected in randomized identification format without asking for personal information, such as names or email addresses. Participants received a reward upon completion based on their registration status in the Surveroid database. A survey request was sent to 8809 individuals via email. A total of 512 (5.8%) responses were received over 5 days of recruitment. Of these, 35 (6.3%) responded that they did not obtain health-related information and were therefore excluded from the study. The final number of participants was 477 (5.4%).

### Ethical Considerations

The Medical Ethics Committee of Kyoto University, Japan, approved this study (#R3215).

### Measures

The online questionnaire included 4 components or groups of items, which required 5 minutes for completion: (1) sociodemographic indicators and experiences during the COVID-19 pandemic, (2) sources of health-related information, (3) health literacy, and (4) COVID-19 knowledge questions.

#### Sociodemographic Indicators

Participants were asked about their sex, age, and education level.

#### Sources of Health-Related Information

Participants were provided with a list of 13 information sources in multiple-response format. They were asked to select the sources they regularly used to obtain health-related information. The list of 13 information sources was compiled from previous studies conducted in Japan and the items used in surveys conducted by the Ministry of Internal Affairs and Communications [11,12,24]. The 13 information sources are listed in Textbox 1.

**List of 13 information sources.**

1. Television (TV)
2. Radio
3. Newspaper
4. Publications (eg, magazines)
5. Municipal newsletters
6. Websites (eg, government and medical manufactures)
7. Web search
8. News apps
9. Video sites (eg, YouTube)
10. Social networking services (SNSs, eg, Twitter, Instagram, and Facebook)
11. Hospitals and pharmacies
12. Family
13. Friends

#### Health Literacy

Health literacy was assessed using the Communicative and Critical Health Literacy (CCHL) scale developed by Ishikawa et al [29]. Health literacy comprises 3 components: functional, interactive, and critical [30]. Functional literacy refers to basic reading and writing skills. Interactive literacy refers to advanced cognitive and literacy skills that can be used to actively participate in daily life, extract information from various forms of communication, understand the meaning, and apply new information to changing situations. Critical literacy refers to more advanced cognitive abilities that can be applied to critically analyze and apply information to successfully control a situation. The CCHL is a self-administered questionnaire that evaluates communicative and critical health literacy among the public. It consists of 5 questions rated on a 5-point scale from 1 (strongly disagree) to 5 (strongly agree). The mean score for all questions is calculated, with a higher mean score indicating higher ability. In a previous study conducted in Japan using the CCHL, the mean score was reported to be 3.58-3.7 [29,31].

#### COVID-19 Knowledge Questions

Participants were asked whether the information about COVID-19 presented in the questions was correct or incorrect. The questions were based on a selection of misinformation that was prevalent in Japan and clearly listed as incorrect on the question and answer (Q&A) page related to COVID-19 provided by the Ministry of Health, Labor and Welfare [32,33]. Participants responded to the questions as correct, unknown, or incorrect. The total number of correct answers was counted as the correct answer score (CAS). Higher scores indicated a higher number of correct answers and greater knowledge of COVID-19, as presented in this study (Table 1).

**Table 1:** COVID-19 knowledge questions.

Question	Answer
COVID-19 is vulnerable to heat, and low-temperature water (25-35°C) has a bactericidal effect.	Incorrect
Alcohol disinfection is effective against COVID-19.	Correct
COVID-19 vaccine makes you infertile.	Incorrect
Vaccination can lead to infection with COVID-19.	Incorrect
The vaccine can be given during pregnancy, during lactation, or while planning a pregnancy.	Correct
If a vaccinated person becomes infected with a mutated virus, they are likely to become seriously ill.	Incorrect

### Statistical Analysis

Participant characteristics were analyzed using descriptive statistics. Participant characteristics and the CCHL score or the CAS were compared using Wilcoxon and Kruskal-Wallis rank sum tests. Concerning information sources, the percentage of participants for each item was calculated (number of responses/total number of participants). The Spearman rank correlation test was used to calculate the correlation coefficient between health literacy, the CAS, and the number of information sources used.

**Figure 1:**
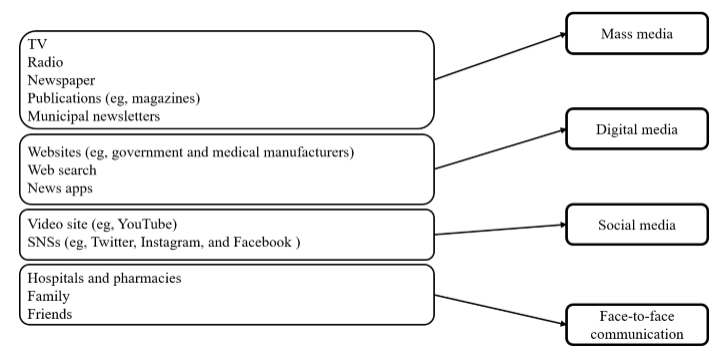
Method of classifying sources of information. SNS: social networking service; TV: television.

The 13 information sources were categorized into 4 media types: mass, digital, social, and face-to-face communication (Figure 1). For example, if participants used TV, radio, and video sites, they were considered to be using mass media and social media. Chi-square tests were used to compare the percentages of those who selected each medium according to sex and age. Multiple linear regression analysis was performed to examine whether the any of the 4 media types had an impact on the CCHL score and the CAS. The dependent variables were the CCHL score and the CAS, the independent variables were the four types of media, and the adjusted variables were sex and age. Each medium was set as 1 for use and –1 for no use, and sex was set as 1 for males and –1 for females. When there was a correlation between the dependent variables, multicollinearity (variance inflation factor [VIF]) was examined. There was no multicollinearity if the VIF<10. The significance level for rejection of the null hypothesis was 5%. Statistical analysis was performed using JMP Pro version 15.0 statistical software (SAS Institute Japan Co.).

## Results

### Participants’ Characteristics

The mean age of the participants was 44.8 (SD 14.3) years. In the comparison of the CCHL score and the CAS by age group, sex, and education level, there was a significant difference only in the CAS by age group; the CAS in the age group of 60-69 years was significantly higher than that in other age groups (Table 2).

**Table 2:** Participants’ characteristics and comparison of the CCHL^a^ score and the CAS^b^.

Variables		Participants, n (%)	CCHL score, mean (SD)	CAS, mean (SD)	
Total		477 (100)	3.61 (0.67)	3.75 (2.01)	
**Age (years); CCHL *P*=.51, CAS *P*<.001^c^**					
	20-29	90 (18.9)	3.57 (0.07)	3.31 (0.20)	
	30-39	92 (19.3)	3.54 (0.07)	3.55 (0.20)	
	40-49	98 (20.5)	3.59 (0.07)	3.26 (0.20)	
	50-59	97 (20.3)	3.67 (0.07)	3.88 (0.20)	
	60-69	100 (21.0)	3.67 (0.07)	4.67 (0.20)^d^	
**Sex; CCHL *P*=.46, CAS *P*=.84**					
	Male	240 (50.0)	3.63 (0.04)	3.74 (0.13)	
	Female	237 (50.0)	3.59 (0.04)	3.76 (0.13)	
**Education level; CCHL *P*>=.09, CAS *P*=.30**					
	Middle school	8 (1.7)	3.28 (0.24)	3.00 (0.71)	
	High school	150 (31.4)	3.54 (0.05)	3.51 (0.16)	
	Technical school and junior college	117 (24.5)	3.62 (0.06)	4.00 (0.19)	
	University	181 (38.0)	3.65 (0.05)	3.77 (0.15)	
	Graduate school	21 (4.4)	3.91 (0.14)	4.14 (0.44)	

^a^CCHL: Communicative and Critical Health Literacy.

^b^CAS: correct answer score.

^c^*P*<.05.

^d^The CAS in the age group of 60-69 years was significantly higher than in other age groups.

### Percentage of Participants by Information Source

**Figure 2:**
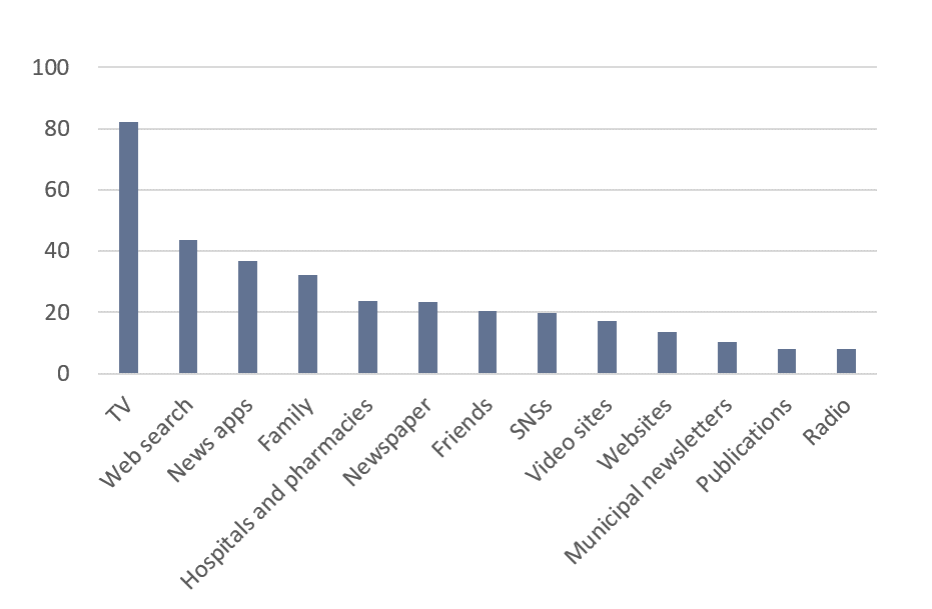
Percentage of responses for information sources. SNS: social networking service; TV: television.

As shown in Figure 2, 392 (82.2%) of the 477 participants received information from the TV. Web searches (n=208, 43.6%) and news apps (n=175, 36.7%) were the next most popular sources. Social networking services (SNSs) and video sites were used by 94 (19.7%) and 82 (17.2%) participants, respectively. Municipal newsletters were used by 49 (10.3%) participants, while publications and radio were used by 39 (8.2%) and 38 (8.0%) participants, respectively.

### Correlation Between the CCHL Score, the CAS, and the Number of Information Sources Used

The CCHL score and the CAS were significantly and positively correlated (ρ=0.12, *P*<.001). Additionally, the number of information sources used was significantly and positively correlated with the CCHL score (ρ=0.22, *P*<.001) and the CAS (ρ=0.19, *P*<.001).

### Types of Media Associated With Health Literacy and COVID-19 Knowledge

Table 3 shows the percentages for all participants, sexes, and age groups when the selected information sources were categorized into different media types. The largest percentage of participants indicated mass media as their source of information, followed by digital media, face-to-face communication, and social media. There were no differences in the use of mass media, digital media, or face-to-face communication by age group. Social media use was significantly higher among individuals aged 20-29 years than among other age groups (*P*<.001). Table 4 displays the multiple linear regression analysis results, with the CCHL score as the dependent variable, the 4 types of media as independent variables, and sex and age as adjustment variables. Table 5 displays the multiple linear regression analysis results, with the CAS as the dependent variable, the 4 types of media as independent variables, and sex and age as adjustment variables. In all cases, a VIF<10 was observed. The CCHL score was significantly associated with access to information from digital media (β=.14, *P*=.003), where β is the standardized regression coefficient, and face-to-face communication (β=.11, *P*=.02). The CAS was significantly associated with access to information from mass media (β=.09, *P*=.05), digital media (β=.17, *P*<.001), and face-to-face communication (β=.10, *P*=.02).

**Table 3:** Distribution by media type (N=477).

Media type	Total, n (%)	Males, n (%)	Females, n (%)	*P* value	20-29 years, n (%)	30-39 years, n (%)	40-49 years, n (%)	50-59 years, n (%)	60-69 years, n (%)	*P* value
Mass media	413 (86.6)	206 (85.8)	207 (87.3)	.69	73 (81.1)	78 (84.8)	83 (84.7)	82 (84.5)	97 (97.0)	.42
Digital media	288 (60.4)	144 (60.0)	144 (60.8)	.93	53 (58.9)	55 (59.8)	54 (55.1)	64 (66.0)	62 (62.0)	.78
Social media	144 (30.2)	69 (28.8)	75 (31.7)	.55	50 (55.6)	32 (34.8)	26 (26.5)	16 (16.5)	20 (20.0)	<.001^a,b^
Face-to-face communication	222 (46.5)	95 (39.6)	127 (53.6)	.002^a^	40 (44.4)	39 (42.4)	47 (48.0)	46 (47.4)	50 (50.0)	.73

^a^*P*<.05.

^b^Social media use in the age group of 20-29 years was significantly higher than in the other age groups. Social media use in the age group of 30-39 years was significantly higher than in individuals aged 50-59 and 60-69 years. Individuals aged 40-49 years had significantly higher social media use than individuals aged 50-59 years.

**Table 4:** Multiple linear regression analysis with the CCHL^a^ score as the dependent variable and sex^b^ and age as adjustment variables.

Media type	β^c^	95% CI	*P* value
Mass media	.04	–0.04 to 0.13	.33
Digital media	.14	0.03-0.16	.003^d^
Social media	–.02	–0.09 to 0.05	.63
Face-to-face communication	.11	0.01-0.14	.02^d^

^a^CCHL: Communicative and Critical Health Literacy.

^b^Media use and male sex were set as 1.

^c^Standardized regression coefficient.

^d^*P*<.05.

**Table 5:** Multiple linear regression analysis with the CAS^a^ as the dependent variable and sex^b^ and age as adjustment variables.

Media type	β^c^	95% CI	*P* value
Mass media	.09	0-0.51	.05^d^
Digital media	.17	0.17-0.55	<.001^d^
Social media	–.02	–0.25 to 0.16	.69
Face-to-face communication	.10	0.03-0.39	.02^d^

^a^CAS: correct answer score.

^b^Media use and male sex were set as 1.

^c^Standardized regression coefficient.

^d^*P*<.05.

## Discussion

### Principal Findings

This study examined the following 3 aspects regarding the COVID-19 infodemic: (1) the relationship between health literacy and COVID-19 knowledge and the number of information sources used, (2) the influence of media use on health literacy, and (3) the influence of media use on knowledge of COVID-19. To the best of our knowledge, this is the first study to examine whether access to information from the 4 major types of media sources is associated with health literacy and COVID-19 knowledge during the COVID-19 infodemic. The Spearman rank correlation test revealed a significant positive correlation between health literacy, COVID-19 knowledge, and the number of information sources used. Multiple linear regression analysis revealed that those with higher health literacy access information through digital media and face-to-face communication. Additionally, the more COVID-19 knowledge people had, the more they accessed information from mass media, digital media, and face-to-face communication.

The CCHL scores of the participants in this study were comparable to those reported in previous studies conducted in Japan [29,31]. Furthermore, health literacy was positively correlated with age [34]. In this study, there was no significant difference between age and the CCHL score. However, the higher the age, the higher the CCHL score, indicating a similar trend as in previous studies. The education level tended to be higher than the census results [35]. Thus, the participants in this study may have had a higher level of education than the general Japanese population.

Participants were more likely to access health-related information from mass media. Previous studies conducted outside Japan have reported that the highest percentage of participants used family members and medical professionals, such as primary care providers and nurses, as information sources rather than mass media [12,13,23]. Furthermore, in studies conducted among young adults, the highest percentage of participants used the internet [22,25]. However, studies conducted in Japan have shown that older adults access information from mass media and family members [24] and that younger adults access information from the internet more than older adults [11]. The results of this study showed that TV, web searches, news apps, and family members are the most common information sources, in that order, with medical professionals ranking fifth overall. However, people in Japan may access information from medical professionals less frequently than those outside the country. The average age at which participants accessed information from mass media, digital media, and face-to-face communication was almost the same; however, the average age was 7 years younger for social media. In recent years, the age group using the internet has expanded, with a 2020 survey showing that almost 100% of individuals aged 20-59 years and 80% of individuals aged above 60 years use the internet [11]. However, social media use is more frequent among younger age groups, with over 90% of individuals aged 20-29 years and only about 60% of individuals aged above 60 years using social media [11]. Therefore, the average age of social media users in this study was also considered younger.

Additionally, the results indicated that the higher the health literacy, the greater the COVID-19 knowledge. Prior research has shown that individuals with low health literacy have more difficulty finding and understanding information about COVID-19 than those with high health literacy [21]. This study supports these findings. Moreover, the greater the number of information sources used, the higher the health literacy and COVID-19 knowledge. Prior research has shown that individuals with higher health literacy are more likely to use various information sources [23,24]. Perhaps, individuals may have also gained knowledge about COVID-19 by obtaining information from diverse sources. It is also possible that the more information sources they used, the higher their CCHL scores; the CCHL scale includes an item about whether they obtained information from a variety of information sources.

The study results indicate that the higher the health literacy, the more the information accessed through digital media and face-to-face communication. Previous studies have reported that people with higher health literacy are more likely to access information from websites (especially medical-related websites), family, and friends [12,23]. Therefore, the results of this study are consistent with the previous findings. Digital media includes internet searches and news apps. Information accessed through digital media ranges from highly reliable sources, such as public institutions and medical manufacturers, to a considerable volume of unverified and unreliable sources, such as personal blogs. Previous studies have reported that many web pages appear when individuals search for health information; however, there are gaps in availability, with insufficient or contradictory content [36]. Therefore, to access information through digital media, it is necessary to select the required information from the vast amount of available information using appropriate search terms and to understand and analyze the content. This differs from mass media in that it widely conveys information in a 1-way manner. This process utilizes communicative and critical literacy. Previous research has reported that people with low health literacy underestimate high-quality information and overestimate low-quality information on the web, making it difficult to accurately judge the information [37]. It is more difficult for these individuals to select the necessary information from digital media. Therefore, higher health literacy is associated with digital media use.

Moreover, accessing information through face-to-face communication is consistent with the process of communicative literacy in that information is obtained through communication with various people. Therefore, higher health literacy is associated with accessing information through face-to-face communication.

Additionally, the results indicate that the more COVID-19 knowledge participants had, the more they accessed information from mass media, digital media, and face-to-face communication. Previous studies have shown that the source of fake news and misinformation regarding COVID-19 is often social media [5]. Social media can spread uncertain or low-quality information, which can lead to misinformation [38,39]. Furthermore, social media uses algorithms that link content based on how data are handled and prioritized [40]. Once misinformation is viewed, similar information may appear repeatedly and is assumed to be correct. In contrast, mass and digital media (eg, news sites) have a check system to avoid conveying misinformation and convey true information at the same time as misinformation [11]. Therefore, it may be easier for recipients to judge true information. Notably, social media complies with WHO and global health authorities and provides links to the websites of public institutions during the COVID-19 pandemic and reminds people to access information with a high level of evidence [41]. However, in a survey conducted during the COVID-19 pandemic in Japan, 30% of participants confirmed the authenticity of information they thought was untrue, while 50% did not confirm the authenticity of information [42]. This finding indicates that alerts may not be sufficient to lead people to access evidence-based information. Furthermore, our results suggest that misinformed people may access the media to obtain misinformation on their own. In such cases, directing people to highly evidence-based information may not be sufficient. Therefore, it might be possible to improve the situation by conveying misinformation as well as true information at the same time as mass media and digital media, such as news sites.

Considering the results, both health literacy and COVID-19 knowledge were associated with access to information from digital media and face-to-face communication. Health literacy and COVID-19 knowledge may be improved by providing opportunities to use digital media and face-to-face communication.

Moreover, the study results showed that the younger the age, the less the COVID-19 knowledge and the greater the use of social media. Previous studies have shown that younger people have lower health literacy [34]. Since social media is a major source of misinformation on COVID-19 [5], individuals may disseminate and spread information without proper understanding. Furthermore, it may be important for young adults to improve their health literacy and to be provided with the correct knowledge about COVID-19.

### Limitations

This study was conducted only in Japan; thus, further studies are needed to generalize the results to other countries. The study only included participants registered with Surveroid, with a response rate of 5.8%. Therefore, it is necessary to increase the demographics and the number of participants to strengthen the results of this study. Furthermore, this was a cross-sectional study and causal relationships could not be demonstrated. A longitudinal study would need to be conducted to demonstrate a causal relationship. The COVID-19 knowledge questions used in this study were obtained from the Ministry of Health, Labor and Welfare website. Since other misinformation has circulated in Japan [42], it is necessary to examine other COVID-19 knowledge in the future. Finally, the participants were asked about the information sources they did or did not use. The relationship between health literacy and COVID-19 knowledge can be examined in more detail by asking detailed questions about the frequency of access and priorities.

### Future Perspectives

The COVID-19 pandemic is still ongoing. Therefore, it is crucial to have adequate access to information about COVID-19.

The prevailing misinformation about COVID-19 is changing with time and the type of prevalent virus. The prevalence of misinformation is also likely to vary from country to country. Therefore, it is necessary to generalize this finding by expanding the area and period in which the survey is conducted. Several health literacy scales exist, in addition to the survey instruments used in this study. In particular, a more detailed assessment of social and digital media use may be possible by measuring eHealth literacy. Furthermore, there is a need to increase the level of evidence by conducting longitudinal studies to investigate the frequency of and changes in media use with interventions to improve health literacy.

### Conclusion

This study examined the association between health literacy, COVID-19 knowledge, and information sources during the COVID-19 pandemic in Japan. The results revealed that the higher the health literacy, the more the knowledge about COVID-19, and the more information sources used, the higher the health literacy and the more accurate the COVID-19 knowledge. Individuals with higher health literacy were found to access information through digital media and face-to-face communication, while individuals with more knowledge about COVID-19 accessed information through mass media, digital media, and face-to-face communication. Health literacy and COVID-19 knowledge may be improved using various information sources, especially by providing opportunities to use digital media and face-to-face communication. Furthermore, it may be essential to improve health literacy and provide accurate knowledge about COVID-19 to young individuals. During the ongoing COVID-19 infodemic, it is crucial to determine truthful information and avoid being swayed by misinformation.

## Abbreviations

CAS: Correct Answer Score
CCHL: Communicative and Critical Health Literacy
SNS: social networking service
TV: television
VIF: variance inflation factor
WHO: World Health Organization

## References

[1] World Health Organization Munich Security Conference.

[2] World Health Organization Infodemic.

[3] The Lancet Infectious Diseases (2020). The COVID-19 infodemic. Lancet Infect Dis.

[4] Statista Social Media Use during COVID-19 Worldwide - Statistics & Facts.

[5] Naeem SB, Bhatti R, Khan A (2021). An exploration of how fake news is taking over social media and putting public health at risk. Health Info Libr J.

[6] Ahmed W, Vidal-Alaball J, Downing J, López Seguí F (2020). COVID-19 and the 5G conspiracy theory: social network analysis of Twitter data. J Med Internet Res.

[7] Ahmed W, López Seguí F, Vidal-Alaball J, Katz MS (2020). COVID-19 and the "film your hospital" conspiracy theory: social network analysis of Twitter data. J Med Internet Res.

[8] Rocha YM, de Moura GA, Desidério GA, de Oliveira CH, Lourenço FD, de Figueiredo Nicolete LD (2021). The impact of fake news on social media and its influence on health during the COVID-19 pandemic: a systematic review. Z Gesundh Wiss.

[9] Vosoughi S, Roy D, Aral S (2018). The spread of true and false news online. Science.

[10] Konishi Y, Saito T, Ishikawa T, Kanai H, Igei N (2021). How did Japan cope with COVID-19? Big data and purchasing behavior. Asian Econ Pap.

[11] Ministry of Internal Affairs and Communications Information and Communications in Japan. White Paper 2021.

[12] Chen X, Hay JL, Waters EA, Kiviniemi MT, Biddle C, Schofield E, Li Y, Kaphingst K, Orom H (2018). Health literacy and use and trust in health information. J Health Commun.

[13] Chen X, Orom H, Hay JL, Waters EA, Schofield E, Li Y, Kiviniemi MT (2019). Differences in rural and urban health information access and use. J Rural Health.

[14] Kelley MS, Su D, Britigan DH (2016). Disparities in health information access: results of a county-wide survey and implications for health communication. Health Commun.

[15] Ministry of Internal Affairs and Communications Information and Communications in Japan. White Paper 2014.

[16] Ministry of Internal Affairs and Communications Information and Communications in Japan. White Paper 2015.

[17] Kaplan AM, Haenlein M (2010). Users of the world, unite! The challenges and opportunities of social media. Bus Horiz.

[18] Abel T, McQueen D (2020). Critical health literacy and the COVID-19 crisis. Health Promot Int.

[19] Chong YY, Cheng HY, Chan HYL, Chien WT, Wong SYS (2020). COVID-19 pandemic, infodemic and the role of eHealth literacy. Int J Nurs Stud.

[20] Nutbeam D, Muscat D (2021). Health promotion glossary 2021. Health Promot Int.

[21] McCaffery K, Dodd R, Cvejic E, Ayrek J, Batcup C, Isautier JMJ, Copp T, Bonner C, Pickles K, Nickel B, Dakin T, Cornell S, Wolf MS (2020). Health literacy and disparities in COVID-19-related knowledge, attitudes, beliefs and behaviours in Australia. Public Health Res Pract.

[22] Schäfer M, Stark B, Werner AM, Tibubos AN, Reichel JL, Pfirrmann D, Edelmann D, Heller S, Mülder LM, Rigotti T, Letzel S, Dietz P (2020). Health information seeking among university students before and during the corona crisis—findings from Germany. Front Public Health.

[23] Fagnano M, Halterman JS, Conn KM, Shone LP (2012). Health literacy and sources of health information for caregivers of urban children with asthma. Clin Pediatr (Phila).

[24] Kinjo H, Ishi K, Saito T, Nomura N, Hamada A (2017). A survey on how older adults access medical and health information and what kinds of problems they face in accessing it. Jpn J Gerontol ?.

[25] Olaimat AN, Aolymat I, Shahbaz HM, Holley RA (2020). Knowledge and information sources about COVID-19 among university students in Jordan: a cross-sectional study. Front Public Health.

[26] Li X, Liu Q (2020). Social media use, eHealth literacy, disease knowledge, and preventive behaviors in the COVID-19 pandemic: cross-sectional study on Chinese netizens. J Med Internet Res.

[27] Su Y (2021). It doesn’t take a village to fall for misinformation: social media use, discussion heterogeneity preference, worry of the virus, faith in scientists, and COVID-19-related misinformation beliefs. Telemat Inform.

[28] Bridgman A, Merkley E, Loewen PJ, Owen T, Ruths D, Teichmann L, Zhilin O (2020). The causes and consequences of COVID-19 misperceptions: understanding the role of news and social media. HKS Misinfo Rev.

[29] Ishikawa H, Nomura K, Sato M, Yano E (2008). Developing a measure of communicative and critical health literacy: a pilot study of Japanese office workers. Health Promot Int.

[30] Nutbeam D (2006). Health literacy as a public goal: a challenge for contemporary health education and communication strategies into the 21st century. Health Promot Int.

[31] Kimura N, Kobayashi T (2020). [Association of health literacy with hypertension, diabetes, and dyslipidemia: a cross-sectional survey of a regional Japanese community]. Nihon Koshu Eisei Zasshi.

[32] Ministry Health, Labor and Welfare Coronavirus (COVID-19).

[33] Ministry Health, Labor and Welfare COVID-19 Vaccination Information.

[34] Nakayama K, Osaka W, Togari T, Ishikawa H, Yonekura Y, Sekido A, Matsumoto M (2015). Comprehensive health literacy in Japan is lower than in Europe: a validated Japanese-language assessment of health literacy. BMC Public Health.

[35] Ministry of Internal Affairs and Communications Census 2015 (in Japanese).

[36] Berland GK, Elliott MN, Morales LS, Algazy JI, Kravitz RL, Broder MS, Kanouse DE, Muñoz J A, Puyol J, Lara M, Watkins KE, Yang H, McGlynn EA (2001). Health information on the internet: accessibility, quality, and readability in English and Spanish. JAMA.

[37] Diviani N, van den Putte B, Giani S, van Weert JC (2015). Low health literacy and evaluation of online health information: a systematic review of the literature. J Med Internet Res.

[38] Patrick M, Venkatesh RD, Stukus DR (2022). Social media and its impact on health care. Ann Allergy Asthma Immunol.

[39] Moorhead SA, Hazlett DE, Harrison L, Carroll JK, Irwin A, Hoving C (2013). A new dimension of health care: systematic review of the uses, benefits, and limitations of social media for health communication. J Med Internet Res.

[40] Hamamreh R, Awad S (2017). Tag ranking multi-agent semantic social networks.

[41] Zarocostas J (2020). How to fight an infodemic. Lancet.

[42] Ministry of Internal Affairs and Communications Report on Information Circulation Survey on New Coronavirus Infections.

